# Integrated Datasets of Proteomic and Metabolomic Biomarkers to Predict Its Impacts on Comorbidities of Type 2 Diabetes Mellitus

**DOI:** 10.2147/DMSO.S244432

**Published:** 2020-07-07

**Authors:** Amrita K Cheema, Prabhjit Kaur, Amina Fadel, Noura Younes, Mahmoud Zirie, Nasser M Rizk

**Affiliations:** 1 Department of Oncology, Lombardi Comprehensive Cancer Center at Georgetown University Medical Center, Washington, DC, USA; 2 Biomedical Sciences Department, College of Health Sciences and Biomedical Research Center, QU Health, Qatar University, Doha, Qatar; 3 Clinical Chemistry Lab, Hamad Medical Corporation, Doha, Qatar; 4 Endocrine Department, Hammad Medical Corporation, Doha, Qatar; 5 Physiology Department, Mansoura Faculty of Medicine, Mansoura, Egypt

**Keywords:** type 2 diabetes mellitus, pathway analysis, regulators, biomarkers, disorders, bioinformatics

## Abstract

**Objective:**

The objective of the current study is to accomplish a relative exploration of the biological roles of differentially dysregulated genes (DRGs) in type 2 diabetes mellitus (T2DM). The study aimed to determine the impact of these DRGs on the biological pathways and networks that are related to the associated disorders and complications in T2DM and to predict its role as prospective biomarkers.

**Methods:**

Datasets obtained from metabolomic and proteomic profiling were used for investigation of the differential expression of the genes. A subset of DRGs was integrated into IPA software to explore its biological pathways, related diseases, and their regulation in T2DM. Upon entry into the IPA, only 94 of the DRGs were recognizable, mapped, and matched within the database.

**Results:**

The study identified networks that explore the dysregulation of several functions; cell components such as degranulation of cells; molecular transport process and metabolism of cellular proteins; and inflammatory responses. Top disorders associated with DRGs in T2DM are related to organ injuries such as renal damage, connective tissue disorders, and acute inflammatory disorders. Upstream regulator analysis predicted the role of several transcription factors of interest, such as STAT3 and HIF alpha, as well as many kinases such as JAK kinases, which affects the gene expression of the dataset in T2DM. Interleukin 6 (IL6) is the top regulator of the DRGs, followed by leptin (LEP). Monitoring the dysregulation of the coupled expression of the following biomarkers (TNF, IL6, LEP, AGT, APOE, F2, SPP1, and INS) highlights that they could be used as potential prognostic biomarkers.

**Conclusion:**

The integration of data obtained by advanced metabolomic and proteomic technologies has made it probable to advantage in understanding the role of these biomarkers in the identification of significant biological processes, pathways, and regulators that are associated with T2DM and its comorbidities.

## Plain Summary

In summary, the incorporation of metabolomic and proteomic data through integrative pathway analysis using different tools would help in understanding the role of various biomarkers in the identification of the biological processes, pathways, upstream regulators, and pathophysiology that are associated with Type 2 Diabetes Mellitus (T2DM) and comorbidities. Therefore, such a study could help to recognize those patients at higher risk for a specific complication and its response to a particular class of anti-diabetic drugs. This study could help in personalized medicine for T2DM.

## Introduction

Recent decades had indicated a remarkable upsurge in the prevalence of diabetes mellitus (DM) worldwide, particularly of type 2 diabetes.^1^ Type 2 Diabetes Mellitus (T2DM) is the most diagnosed form of diabetes characterized by insulin resistance, impaired β-cell function, hyperglycemia, and some comorbidities, including obesity and cardiovascular disease.^2^ The potential impact of diabetes on health, health care system, financial cost, and life expectancy increases in the upcoming years. Optimum treatment of T2DM requires a set of potentially multiple measures to manage hyperglycemia, hyperlipidemia, and to address the risk factors for the array of diabetic complications.

Identification of biomarkers for T2DM and its complications is a challenging issue because of the diverse nature of this disease. Different factors contribute to the heterogenicity of type DM such as the glycemic control, treatment response, duration of the diseases, age of onset and biochemical profile, body mass index, and variations in environmental exposures, which could affect the disease diversity.^3^ Biomarkers are needed for the evaluation of chemical profiles, disease status, target validation, and treatment regimens. Advances in proteomics and metabolic profiling have increased the screening for experimental biomarkers. Serum biomarkers currently exist for T2DM, but it remains a challenge to evaluate pathophysiology on a patient by patient basis. Therefore, novel biomarkers based on the integration of different profiles such as metabolomics, proteomics, and transcriptomics would better reflect the regulation of gene expression and the biologic process in diabetes for preventative strategies and lessen the complications are needed.

Metabolites symbolize intermediate and end products of metabolic pathways that reflect the physiology and dysfunctions of metabolic processes and disorders. Recent technology allows for the assessment of metabolites opening new opportunities to study changes in biochemical pathways for insight into the biological mechanisms of disorders such as T2DM and its comorbidities.^4^ This integration constitutes the promise of personalized medicine (PM).

The personalized medicine could help in the screening of subjects at risk of developing T2DM, as well as one or all of the complicating morbidities associated with microangiopathies, such as retinopathy, neuropathy, nephropathy, and macroangiopathy or large-vessel disease. They also have the potential to direct treatment planning, regarding personalized goal setting, choice of treatments, and treatment prioritization.^5^

In this study, the aim is to accomplish a comparative and integrative investigation of metabolomic and proteomic datasets of gene expression to identify differentially regulated genes (DRGs) as potential predictive biomarkers associated with type 2 diabetes and its complications. The integration of the dataset of DRGs is used to reveal significant pathways and biological functions and diseases that are relevant to understand the pathogenesis of associated comorbidities of diabetes and its complications. The study pursued to recognize biological processes and metabolic pathways of DRGs, which are interrelated to T2DM that were differentially up- or downregulated in comparison to healthy controls. Therefore, the overall target of the current study is to recognize those patients at higher risk for a disorder or complication associated with T2DM through understanding the biological networks and pathways underlie these diseases that could respond better to management and drug treatment.

## Methodology

### Study Selection and Sample Collection

Datasets from two previously published studies on T2DM by our group were selected for gene expression integration.^5^,^6^ Each gene was described by fold change. All data was collected from serum/plasma samples of patients at the Hamad Medical Hospital, Qatar (HMC) with T2DM versus a healthy control group, all subjects were unrelated Arab subjects of different countries to ensure lack of inheritance. A total of 140 subjects were involved in the present study, of which 85 T2DM subjects and 55 healthy controls, non-diabetics. T2DM was diagnosed by the medical team of the diabetic unit (HMC) according to the American Diabetes Association (ADA) criteria, consisting of fasting plasma glucose ≥ 126 mg/dL (≥6.993 mmol/L), 2 hr. plasma glucose ≥200 mg/dL (11.1 mmol/L) during an oral glucose tolerance test and/or HbA1C ≥ 6.5%.^7^ The diagnostic criteria were based on the diagnostic standards based on oral GTT following ADA diagnostic criteria to be sure that all study subjects, including the controls, are not pre-diabetic or diabetic. The age of diabetic patients was older than the age of the control, but this was accounted for in the current study as we compared the data using AUC, and a Supplementary Table 1 was provided. The study was approved by the Institutional Review Board of the Hamad Medical Corporation, Qatar University, and Georgetown University (HMC approval number 8249/08, QU-IRB-06/09 and 2008–538, respectively). The study was performed according to the principles expressed in the Declaration of Helsinki. Written informed consent was obtained from each subject after a full explanation of the purpose, nature, and risk of all procedures used.

After overnight fasting, venous blood was collected as previously described.^5^ Serum, plasma, and buffy coat were separated from the whole blood and stored at −80ºC within 4 hours of collection. For maximum longevity and to avoid repeated freeze-thaw cycles, the plasma, and serum samples were aliquoted extensively and stored at −80ºC till further use.^5^

For the metabolomic and lipidomic profiling experiment levels of high-density lipoprotein cholesterol, total cholesterol, and triglycerides were assayed by automated clinical laboratory methods using a diagnostic analyzer. Low density lipoprotein cholesterol levels were estimated using the Friedewald formula: LDL-C = TC – HDL-C (TG/5).^8^ Serum aminotransferase, albumin, alkaline phosphatase, and creatinine were also assayed using a diagnostic analyzer at HMC as previously published.^9^,^10^ Metabolite extraction from plasma was done by adding 175 µL of 66% acetonitrile (in water) containing internal standards to 25 µL of plasma. The samples were incubated on ice for 15 minutes and centrifuged at 14,000 rpm at 4ºC for 20 minutes. The supernatant was transferred to a fresh tube and dried under a vacuum. The dried samples were resuspended in 100 µL of solvent A (98% water and 2% acetonitrile) for UPLC-ESI-Q-TOF-MS analysis. In order to increase metabolome coverage, plasma lipidomics was performed by extracting lipids using the method described by.^11^

For the protein expression profiling experiment, serum samples were delipidated according to the protocol described by Cham and Knowles in preparation for iTRAQ analysis.^12^ ProteoExtract Albumin/IgG (from Calbiochem), and Vivapure anti-HSA-IgG kits were used to evaluate the efficiency of high abundance protein depletion from serum samples. Total protein concentration was calculated by the Bradford Assay. The Vivapure anti-HSA/IgG kit was used for the iTRAQ experiment.

### Data Processing and Metabolite Identification

Centroided and integrated UPLC-TOFMS data were pre-processed using the XCMS software and normalized to the ion intensity of the respective internal standards for the metabolomic and lipidomic profiling experiment.^13^ Multivariate analyses were performed to delineate significantly altered metabolites. The metabolites were identified via accurate mass-based search using the Madison Metabolomics Consortium Database (MMCD),^14^ the Human Metabolome Database (HMDB),^15^ and LIPID MAPS. The lipids with significant fold change in T2DM as compared to the control group were identified via a spectral matching based lipid identification software, SimLipid v 3.0 and LIPID MAPS,^16^ and confirmed against fragmentation pattern of standards. Metabolite identifications confirmed by comparing the retention time under the same chromatographic conditions and by matching the fragmentation pattern of the parent ion from the biological sample to that of the standard metabolite using tandem mass spectrometry (UPLC-TOFMS/MS).

### Nano-UPLC-MS/MS and iTRAQ Analysis

Nano UPLC-MS/MS analysis was conducted by an electrospray quadrupole time of flight (ESI-QTOF) mass spectrometer coupled with a Nano-Acquity-UPLC system. Relative abundance quantitation, peptide, and protein identifications were performed using Protein Pilot software 3.0 (ABSCIEX). Data were analyzed with MMTS as a fixed modification of cysteine, and the database was searched with a 95% confidence interval rate for protein identifications. High confidence peptides of the target proteins exhibiting rich production spectrum were selected for multiple reaction monitoring (MRM) assays. MRM data were processed using TargetLynx 2.0, while Graph Pad Prism program v 5.0 was used for statistical analysis and to generate the receiver operating characteristics. The Wilcoxon test was used for the comparison of each peptide.

### Luminex Analysis

The serum samples of the study subjects (Diabetics and controls) were used to evaluate the different panels of biomarkers, including the inflammatory, adipokines, oxidative stress, metabolic, CVD, and bone markers. The following kits were used to evaluate such different panels, including the following multiplex assays, HMHMAG-34, HCVD1-67AK, APOMAG-62K, HADK1MAG-61k-03, HBN1A‐51K, and HCVD2MAG-67K. All assays were performed in triplicates according to the manufacturer’s instructions from Millipore (Merck Millipore, Billerica, MA, USA). The assays were performed using a Luminex200 (Austin, TX, USA). Of note, PCA analysis was performed for all dataset of the study to obtain DRGs as we previously published.^5^,^6^

### Statistical Analyses

Clinical and biochemical data are expressed as mean ± SD. All statistical analyses were performed using the SPSS program for Windows (version 21 statistical software: Texas instruments, IL, USA). Differences between control and T2DM were performed using Student’s *t*-test or Mann–Whitney/Wilcoxon when appropriate. ANCOVA was used to perform analysis, including the age to compare the biochemical data of the study subjects and AUC (see Supplementary Table 1). Two-tailed p value is significant when p < 0.05.

### Bioinformatic Analysis

Ingenuity Pathway Analysis (IPA; http://www.ingenuity.com/) was performed to identify canonical pathways, diseases and functions, and gene networks that are most significant to the dataset and to categorize differentially dysregulated genes in specific diseases and functions. We also used Pathway Studio 9 (Elsevier) for Integrated pathway mapping. For Network generation, a data set containing gene/protein/metabolite identifier and corresponding fold change and UniProt ID was uploaded into the application. Each protein/metabolite ID was mapped to its corresponding gene/protein/metabolite in the Ariadne ResNet Mammalian database. The metabolites were grouped based on their fold change and used to develop networks based on regulation and connectivity.

## Results

### Clinical Data of the Study Subjects

The characteristics of the study population are described in Table 1. As shown in Table 1, diabetic subjects have significantly higher values for age, BMI, glucose,  HbA_1_c%, estimated average glucose (eAC), triglycerides, ALT, and CRP than control healthy subjects. Diabetic subjects have significantly lower values for C-peptide and insulin than healthy control subjects. Other variables are not significantly different between the two studied groups.

**Table 1:** Clinical and Biochemical Data of the Study Subjects

Characteristics	Control (n=55)	T2DM (n=85)	P
Age (years)	35.69 (11.11)	52.35(9.96)	<0.0001
BMI (kg/m^2^)	29.12 (5.26)	31.28 (5.11)	0.021
Glucose (mM)	4.97 (1.12)	8.67(3.73)	<0.0001
HbA_1_c (%)	5.71 (0.62)	7.56(1.83)	<0.0001
eAG (mM)	6.50	9.50	<0.0001
Triglycerides (mM)	1.21 (0.78)	1.66 (1.11)	0.010
TC (mM)	4.60 (0.97)	4.83(2.05)	0.448
HDL (mM)	1.62 (0.37)	1.21 (0.39)	0.055
LDL (mM)	2.72(0.76)	2.82(0.75)	0.424
Albumin (g/L)	45.56(2.63)	45.24(2.78)	0.524
ALT (U/L)	17.11(2.42)	27.22(1.99)	0.002
Total bilirubin (µmol/l)	7.96 (3.85)	9.32(6.19)	0.128
Total proteins(g/L)	73.17(7.56)	71.49(9.32)	0.294
ALP (U/L)	72.31(31.33)	71.25(23.39)	0.827
Creatinine (µmol/l)	69.02 (15.64)	72.68(23.91)	0.349
CRP (mg/l)	5.63 (2.09)	7.78 (7.11)	0.046
C-peptide (pg/mL)	348.85 (173.79)	243.08(127.59)	<0.0001
Insulin (pg/mL)	294.88 (75.45)	99.75 (85.29)	<0.0001

**Notes:** Data are presented as means (SD). ANCOVA test was used to analyze, including age and gender effects. Two tailed p value is significant at <0.05. **Abbreviations:** BMI, body mass index; SBP, systolic blood pressure; DSB, diastolic blood pressure; TC, total cholesterol; TG, triglycerides; LDL-C, low-density lipoprotein cholesterol; HDL-C, high-density lipoprotein cholesterol; ALT, alanine transferase; ALP, alkaline phosphatase; eAG, estimated average glucose; HbA_1_c, glycosylated hemoglobin; CRP, c-reactive protein.

### Differentially Expressed Gene Analysis

Table 2 displays 94 differentially expressed proteins, of note few proteins (9) are downregulated, while the majority (85) are up-regulated, as shown in Table 2.

**Table 2:** T2DM Differentially Expressed Proteins

Metabolite/Protein	Name	(GenBank ID)	Fold Change (T2DM to Control)	P value
	(Gene Symbol)	Accession Number		
Insulin	INS	P01308	−1.73	0.025
Matrix metallopeptidase 9	MMP9	P14780	−1.9	0.004
Secreted phosphoprotein 1	SPP1	P10451	−2.66	0.031
Islet amyloid polypeptide	IAPP	P10997	−2.45	0.002
Apolipoprotein C3	APOC3	P02656	−0.62	0.015
Apolipoprotein H	APOH	P02749	−1.43	0.024
Apolipoprotein C1	APOC1	P02654	−1.55	0.032
Apolipoprotein A2	APOA2	P02652	−1.65	0.014
Immunoglobulin heavy constant gamma 4	IGHG4	P01861	1.24	0.021
Tumor necrosis factor	TNF	P01375	1.62	0.006
Intercellular adhesion molecule 1	ICAM1	Q99930	1.75	0.004
Angiotensinogen	AGT	P01019	2.45	0.001
Coagulation factor II	F2	P00734	2.55	0.0001
Kininogen 1	KNG1	P01042	3.16	0.002
Interleukin 6	IL6	P05231	2.44	0.021
Parathyroid hormone	PTH	P01270	1.92	0.035
Apolipoprotein E	APOE	P02649	1.85	0.018
Glucagon	GCG	P01275	2.25	0.009
Gelsolin	GSN	P06396	1.65	0.016
Chemokine (C-C motif) ligand 2	CCL2	P13500	2.8	0.024
Complement component 5	C5	P01031	1.85	0.031
Leptin	LEP	P41159	2.51	0.0001
Serpin peptidase inhibitor, clade E, member 1	SERPINE1	P05121	−1.8	0.042
Fibronectin 1	FN1	P02751	1.84	0.025
Vitronectin	VTN	P04004	1.48	0.035
Apolipoprotein A-IV	APOA4	P06727	1.95	0.018
Apolipoprotein A-I	APOA1	P02647	2.15	0.029
Transferrin	TF	P02787	2.24	0.016
Peptide YY	PYY	P10082	1.48	0.041
Ghrelin/obestatin prepropeptide	GHRL	Q9UBU3	2.58	0.026
Resistin	RETN	Q9HD89	1.95	0.013
Paraoxonase 1	PON1	P27169	2.36	0.024
Retinol binding protein 4	RBP4	P02753	3.75	0.001
Titin	TTN	Q8WZ42	1.82	0.04
Plasminogen	PLG	P00747	3.85	0.035
Complement component 3	C3	P01024	2.5	0.018
Actin, gamma 1	ACTG1	P63261	1.24	0.027
Transthyretin	TTR	P02766	2.25	0.011
Ceruloplasmin	CP	P00450	3.55	0.009
Serpin peptidase inhibitor, clade F, member 1	SERPINF1	P36955	2.08	0.043
Serpin peptidase inhibitor, calde G, member 1	SERPING1	P05155	1.85	0.038
Apolipoprotein C-III	APOC3	P02656	2.54	0.027
Serpin peptidase inhibitor, clade C member 1	SERPINC1	P01008	1.69	0.014
Alpha-1-microglobulin/bikunin precursor	AMBP	P02760	1.25	0.026
Apolipoprotein B	APOB	P04114	3.21	0.018
Glutamic-pyruvate transaminase	GPT	P24298	4.21	0.004
Haptoglobin	HP	P00738	3.24	0.003
Kallikrein B, plasma 1	KLKB1	P03952	3.25	0.01
Serpin peptidase inhibitor, clade A, member 6	SERPINA6	P08185	2.75	0.027
Clusterin	CLU	P10909	2.65	0.042
Melanocortin 2 receptor	MC2R	Q01718	4.2	0.057
Complement factor B	CFB	P00751	2.55	0.048
Orosomucoid 1	ORM1	P02763	2.85	0.034
Alpha-2-glycoprotein 1, zinc-binding	AZGP1	P25311	3.05	0.017
Alpha-2-HS-glycoprotein	AHSG	P02765	2.45	0.027
Coagulation factor IX	F9	P00740	4.15	0.0001
Serum Amyloid A4	SAA4	P35542	3.4	0.0001
Apolipoprotein A-II	APOA2	P02652	2.7	0.020
Serpin peptidase inhibitor, clade F, member 2	SERPINF2	P08697	1.69	0.032
Apolipoprotein A-II	APOC2	P02655	1.68	0.014
Keratin 14	KRT14	P02533	3.05	0.007
Serpin peptidase inhibitor, clade A, member 3	SERPINA3	P01011	2.45	0.001
Pro-platelet basic protein	PPBP	P02775	4.6	0.007
Serpin peptidase inhibitor, clade D, member 1	SERPIND1	P05546	3.51	0.003
Complement component 1, s subcomponent	C1S	P09871	2.45	0.028
Complement component 4, binding protein, alpha	C4BPA	P04003	1.85	0.037
Lectin, galactoside-binding, soluble, 3 binding protein	LGALS3BP	Q08380	2.05	0.024
Proteoglycan 4	PRG4	Q92954	1.45	0.036
Histidine-rich glycoprotein	HRG	P04196	1.65	0.038
Fibulin 1	FBLN1	P23142	1.58	0.044
Lumican	LUM	P51884	1.95	0.043
Apolipoprotein L, 1	APOL1	O14791	1.25	0.03
Complement component 4B	C4B	P0C0L5	2.24	0.037
Hemopexin	HPX	P02790	2.48	0.022
Complement component 1,	C1R	P00736	1.47	0.041
r subcomponent				
Group-specific component Vitamin D Binding Protein	GC	P02774	1.56	0.041
Ficolin	FCN3	O75636	2.05	0.035
Inter-alpha inhibitor H3	ITIH3	Q06033	1.84	0.028
Complement component	C2	P06681	2.14	0.037
Leucine-rich alpha-2-glycoprotein 1	LRG1	P02750	3.15	0.021
Haptoglobin-related protein	HPR	P00739	2.85	0.019
Keratin 2	KRT2	P35908	3.48	0.003
Complement factor I	CFI	P05156	3.24	0.008
Immunoglobulin heavy constant gamma 3	IGHG3	P01860	2.15	0.016
Orosomucoid 2	ORM2	P19652	1.85	0.024
Complement component 6	C6	P13671	1.34	0.037
Afamin	AFM	P43652	2.15	0.035
Calmodulin-1	CALM1	P0DP23	3.43	0.010
Alpha-1-B glycoprotein	A1BG	P04217	1.55	0.039
Immunoglobulin kappa constant	IGKC	P01834	1.65	0.026
Complement component 7	C7	P10643	2.31	0.028
Complement component 9	C9	P02748	2.46	0.031
Complement component 1, q subcomponent, C chain	C1QC	P02747	2.81	0.033
Complement component 8, alpha polypeptide	C8A	P07357	1.89	0.029

**Notes:** Data displays the list of the 94 metabolites that were identified in the ResNet Mammalian database with their corresponding GenBank ID. Data represent the differentially expressed regulated proteins with fold changes and p values in T2DM compared to control subjects.

### Bioinformatic Analysis

Data represent the differentially expressed regulated proteins were further evaluated to obtain details of biological processes, cellular functions, networks, and signaling pathways related to comorbidities and diseases associated with T2DM. The dataset was integrated into different software such as IPA software, and Pathway Studio 9 software for the analysis of molecular pathways and networks.

### Biological Pathways

#### Canonical Pathway Analysis

**Figure 1:**
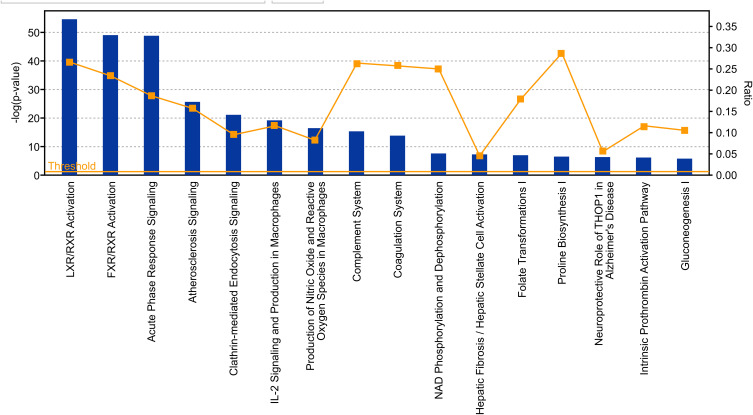
Displays a conical illustration of the top 15 significant enriched pathway analysis in the proteomic and metabolomic dataset, which are differentially expressed genes in T2DM. Data are presented as bars based on the -log of P values and the ratio indicates the percentage of DRGs of the study compared to the IPA knowledge base.

The dataset was analyzed using IPA core analysis to achieve a fundamental profile of the molecular processes underlying T2DM and its complications. Related canonical pathways categorized the differentially expressed genes. Categorization was based on a multiple testing correction of p-value less than 10^−2^ of the present dataset to the numbers of genes of the IPA knowledge data of each pathway. Figure 1 displays a conical illustration of the top 15 significant enriched biological pathways in T2DM patients (see Supplementary Table S2 for details). The top 5 enriched signaling pathways in rank were LXR/RXR Activation, FXR/RXR Activation, Acute Phase Response Signaling, Atherosclerosis Signaling, and Clathrin-mediated Endocytosis, as shown in Table 3.

**Table 3:** Top Five Canonical Pathways of DRGs of the Diabetic Subjects with Their p-values (Based on Core Analysis in Ingenuity Pathway Analysis [IPA])

Top Canonical Pathways			Downregulated Genes	Upregulated Genes
Name	p-value	Overlap		
LXR/RXR activation	2.12^E-55^	26.6%	3/128 (2%)	31/128 (24%)
FXR/RXR activation	5.24^E-50^	23.4%	3/137 (2%)	29/137 (21%)
Acute phase response signaling	1.12^E-49^	18.8%	1/181 (1%)	33/181 (18%)
Atherosclerosis signaling	2.09^E-27^	15.7%	2/127 (2%)	18/127 (14%)
Clathrin-mediated endocytosis signaling	6.83^E-22^	9.7%	2/196 (1%)	17/196 (9%)

**Figure 2:**
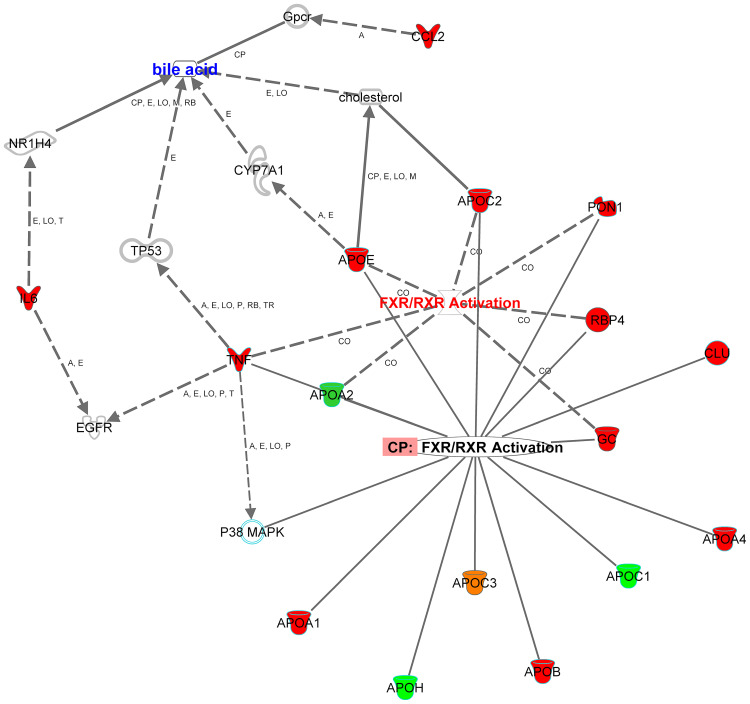
Role of the farnesoid X receptor (FXR) and bile acids. Network displays DRGs, which are upregulated (red) and downregulated (green) genes. The figure shows the role of these DRGs in activation (A), expression (E), correlation (CO), localization (LO), phosphorylation (P), transcription (T), translocation (TR), regulatory binding (RB), inhibition (I), and molecular cleavage (M), which induces the nuclear farnesoid X receptor (FXR) or NR1H4 (nuclear receptor subfamily 1, group H, member 4) with its effect on the suppression of cholesterol 7 alpha-hydroxylase (CYP7A1), the rate-limiting enzymes of bile acid synthesis from cholesterol. **Abbreviation:** CP, canonical pathway.

The Retinoid X Receptors (RXRs) were on top of the signaling pathways, which includes 31 upregulated genes and 3 downregulated genes of the 128, as shown in Supplementary Table S3). RXRs are nuclear receptors that exert the biological effects of retinoids by the participation of retinoic acid-mediated gene, which affects biological functions such as lipid metabolism, molecular transport, small molecule biochemistry. The second pathway is The Farnesoid X receptor (FXR), which includes 29 upregulated genes and 3 downregulated genes of the 137 genes of IPA base knowledge (Table S4). FXR is a member of the nuclear family of receptors and has a fundamental function in the regulation of numerous metabolic pathways, such as bile acid metabolism and its control (Figure 2). The third pathway is the Acute Phase Response Signaling, whereas 34 DRGs of the dataset were detected of which one gene is downregulated, and 33 were upregulated out of the 188 genes of IPA base knowledge (Table S5). This pathway is a cytokine signaling pathway where it is activated by tissue injury, trauma, surgery, cancer, immunologic disorders, and in response to microorganisms as a protective pathway. Following that, the next fourth pathway is the Atherosclerosis Signaling, whereas 20 DRGs of the data set were detected, of which two were downregulated, and 18 were upregulated of the 127 genes of IPA base knowledge (Table S6). This pathway is a specific form of a chronic inflammatory process that functions as a cell to cell signaling and interactions and cellular movement in the cardiovascular system. The next pathway is Clathrin-mediated Endocytosis Signaling, whereas 19 DRGs of the data set were detected, of which 2 genes were downregulated, and 17 were upregulated out of the 196 genes of IPA base knowledge shown in (Table S7). Clathrin-mediated Endocytosis Signaling is involved in endocytosis, which is the principal pathway for the movement of nutrients, hormones, and other signaling molecules from the extracellular into intracellular structures across the plasma membrane. Other canonical biological pathways were IL-12 Signaling and Production in Macrophages pathway, whereas 16 DEGs of the dataset were detected of which one gene was downregulated, and 15 were upregulated of the 136 genes of the IPA base knowledge (Table S2). IL-12 is produced primarily by dendritic cells, macrophages, and monocytes, and affecting Th1 immune response and Th17 cells activation. Following that, the Production of Nitric Oxide and Reactive Oxygen Species in Macrophages, whereas 16 DEGs of the data set were detected of which one gene is downregulated, and 15 were upregulated of the 194 genes of IPA base knowledge as shown (Table S2). This pathway is central to the control of infection by microbes.

### Diseases Associated with DRGs

Using IPA base knowledge, we detected several significant diseases associated with the present dataset of the current study (Table S8 and S9). The top five affected diseases in rank based on higher p values for multiple testing for corrections were presented in Table 4. These disorders are Neurologic Diseases, the Organismal Injury, and Abnormalities, the Psychological Disorders, the Inflammatory Response Disorders, and Metabolic Disorders.

**Table 4:** Top Five Diseases and Disorders of the DRGs of the Present Study with Their p-values (Based on Core Analysis in Ingenuity Pathway Analysis [IPA])

Diseases and Disorders	p-Value Range	# Molecules
Name		
Neurological disease	2.13^E-09^–2.44^E-30^	63
Organismal injury and abnormalities	1.19^E-08^–2.44^E-30^	89
Psychological disorders	1.01^E-08^–2.44^E-30^	49
Inflammatory response	1.20^E-08^–8.76^E-29^	76
Metabolic disease	3.88^E-09^–2.13^E-28^	60

Further, we analyze the top significant disorders associated with each category of these diseases, as shown in Table 5.

**Table 5:** Top Disease Category with Examples of Each of the Present Study with Their p-values and DRGs Involved (Based on Core Analysis in Ingenuity Pathway Analysis [IPA])

Disease Category/Example	p-value	Genes in Dataset
Neurologic and psychological diseases		
Progressive neurological disorders	8.17^E-28^	APOE, SERPING1, INS
Dementia	2.44^E-30^	APOE, INS
Alzheimer’s diseases	6.154^E-28^	APOE, INS
Eating disorder	1.01 ^E-8^	LEP, IL6, GCG, SERPINE1, TNF, INS
Organismal injury and abnormalities		
Amyloidosis	2.13^E-28^	INS, IL6 APOE APOA1
Apoptosis of the endothelial cells	3.51^E-11^	RBP4, KLKB1, KNG1, CCL2, F2, LEP, AGT, IL6, APOA1, IGHG3, ATP, SERPINF1, FN1, ICAM1, TNF, GC, VTN
Damage of the genitourinary system	1.243^E-15^	CFB, AGT,IL6, C4A/C4B, ICAM1, TNF GC.
Inflammatory response		
Degranulation of cells	5.06 ^E-24^	PPBP, CCL2, F2, LEP, GCG, C4A/C4B, TNF
Activation of leukocytes	2.63 ^E-15^	RBP4, KLKB1, KNG1, CCL2, F2, LEP, AGT, IL6, APOA1, IGHG3, ATP, SERPINF1, FN1, ICAM1, TNF, GC, VTN.
Metabolic disorders		
Hypertriglyceridemia	3.60 ^E-13^	APOA2, APOB, APOC3, APOE, IL6, SERPINF1, TNF,INS
		

**Note:** Genes in the data set are predicted to be involved based on measurement.

### Neurologic and Psychological Diseases Category

Among this category, progressive neurological disorders included 3 DRGs of the data set of which APOE and SERPING1 are upregulated, and INS is downregulated, which increases the prediction of the progressive neurological disorders, as shown in Table 5. Dementia is a neurological and psychological disorder that included 2 DRGs of the data set, whereas APOE is upregulated, and the INS is downregulated, which increases the prediction of the prediction to dementia, as shown in Table 5. Alzheimer’s disease is a neurological and psychological disorder that included 2 DRGs of the data set, whereas APOE is upregulated, and the INS is downregulated, which increases the prediction of the prediction to Alzheimer’s diseases as shown in Table 5. Eating disorder is a significant psychological problem in diabetic subjects. As displayed in Table 5. The eating disorder is predicted to increase with six DRGs, which are LEP, IL6, GCG, SERPINE1, TNF, and INS.

### Organismal Injury and Abnormalities Category

Amyloidosis, is one of the Organismal Injury and Abnormalities. As displayed in Table 5, INS, IL6 increases while APOE and APOA1 decrease the prediction of amyloidosis.

Apoptosis of the endothelial cells is one of the Organismal Injury and Abnormalities predicted to be increased with (z-score 3.29). As presented in Table 5, the apoptosis of the endothelial cells is predicted to increase with 12 genes of DRGs, which are PLG, RBP4, KNG1, LEP, SERPINA3, AGT, SERPINE1, LUM, SERPINC1, TNF, and SPP1.

The damage of the genitourinary system is predicted to be increased with (z-score 2.203). As displayed in Table 5, the damage of the genitourinary system is predicted to increase with seven DRGs, which are CFB, AGT, IL6, C4A/C4B, ICAM1, TNF, and GC.

### Inflammatory Response Disorders Category

Degranulation of cells is predicted to be increased with active z-score 2.60. As displayed in Table 5, the degranulation of cells is predicted to increase with seven genes, which are PPBP, CCL2, F2, LEP, GCG, C4A/C4B, and TNF. (Figure S1)

Activation of leukocytes is predicted to be increased with active z-score 2.359. As displayed in Table 5, the activation of leukocytes is predicted to increase with 17 genes of DRGs, which are RBP4, KLKB1, KNG1, CCL2, F2, LEP, AGT, IL6, APOA1, IGHG3, ATP, SERPINF1, FN1, ICAM1, TNF, GC, and VTN.

### Metabolic Disorders Category

Hypertriglyceridemia is on the top of the metabolic disorders and cardiovascular diseases (CVD) associated with T2DM, with Z-score of 2.035. Hypertriglyceridemia is predicted to increase with the following upregulated genes APOA2, APOB, APOC3, APOE, IL6, SERPINF1, TNF, and the downregulated gene INS as shown in Table 5.

Dyslipidemia is a metabolic disorder commonly associated with T2DM with a Z-score of 1.260. Dyslipidemia s predicted to increase with the following genes APOA2, APOB, APOC3, IL6, INS, and TNF, as shown in Table 5. See Supplementary figures (Figures S1).

### Functional Disorders Associated with Dataset in T2DM

Furthermore, we analyzed the common functional disorders which are associated with DRGs of that set using IPA base knowledge. The top 5 functions affected are Cellular compromise, Protein synthesis, Molecular transport, Lipid metabolism, and Small molecule biochemistry. Table 6 Illustrates the genes associated with the top functions.

**Table 6:** Top Five Functions of the DRGs of the Present Study with Their p-values (Based on Core Analysis in Ingenuity Pathway Analysis [IPA])

Molecular and Cellular Functions		
Category of Function	p-value Range	# Molecules/Function
Cellular compromise	2.50^E−09^–8.76^E-29^	42
Protein synthesis	1.64^E-12^–2.30^E-25^	46
Molecular transport	1.08^E−08^–6.29^E-25^	59
Lipid metabolism	9.51^E−09^–1.51^E-24^	55
Small molecule biochemistry	1.08^E−08^–1.51^E-24^	58

**Figure 3:**
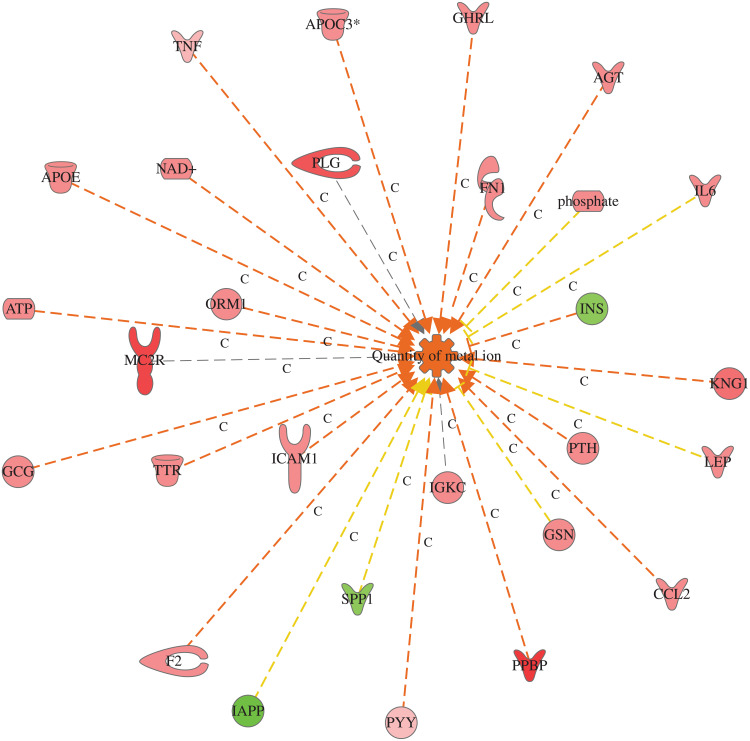
Molecular transport category. Displays DRGs, which are upregulated (red) and downregulated (green) genes involved in molecular transport, quantity of metal ions. Brown arrow, increase prediction; yellow arrow, decreased prediction; and gray arrow, no known predicted effects. RPBP, KNG1, ORM1, CCL2, GHRL, F2, APOC3, AGT, GCG, TTR, PTH, APOE, NAD, ATP, FN1, ICAM1, INS, PYY, and TNF, increase prediction of quantity of metal ions. GCN, SPP1, IAPP, phosphate as IL6 decrease prediction of quantity of metal ions. **Abbreviation:** C, causative relationship.

Further, we explore the top five functions in each category associated with T2DM and identify the genes of the DRGs of the data set per each function, as shown in Table 7. The most affected function in the cellular component category is the degranulation of cells (Table 7) and (Figure S1), of which seven genes are PPBP, CCL2, F2, LEP, GCG, C4A/C4B, TNF increased the prediction of the degranulation. Fat Acid Metabolism under the lipid metabolism category with activation Z-score of 3.62, including 19 genes of the data set increased the prediction of FA metabolism (Figure S2a), Protein synthesis under Protein Metabolism category with a P value of 7.74^E-24^, Z-score of 2.88, with 19 DRGs of the data set to increase the prediction of protein synthesis (Figure S2b). The release of lipids (p=2.11^E-12^), Z-scores of 3.70 having 15 DRGs which increase the prediction of the release of lipids, and release of Ca^+2^, P=1.15^E-11^ and Z-scores of 2.75 having 11 DRGs of the data set which increased the prediction of the release of Ca^+2^, (Figure S2c), and quantity of metal ions (p=1.61^E-20^), Z-scores of 3.16 having 10 DRGs which increase the prediction of the quantity of metal ions (Figure 3) under Molecular transport category. All molecules involved in functions are illustrated in supplementary (Table S10) for details.

**Table 7:** Top Five Functions of Each Category of the Present Study with Their p-values (Based on Core Analysis in Ingenuity Pathway Analysis [IPA])

Top Molecular and Cellular Function per Each Category			
Molecular and Cellular Category/Top Function Example	p-value	z-Score	# Molecules
Cellular compromise/degranulation of cells	5.06 ^E-24^	2.60	42
Protein synthesis/protein metabolism	7.74^E-24^	2.88	43
Molecular transport/release of lipids Molecular transport/quantity of Ca+2	2.11^E-12^ 2.25^E-20^	3.70 3.31	15 27
Lipid metabolism/fat acid metabolism	9.71^E-22^	3.62	32
Small molecule biochemistry/release of Ca^+2^	1.15^E-11^	2.75	14

**Figure 4:**
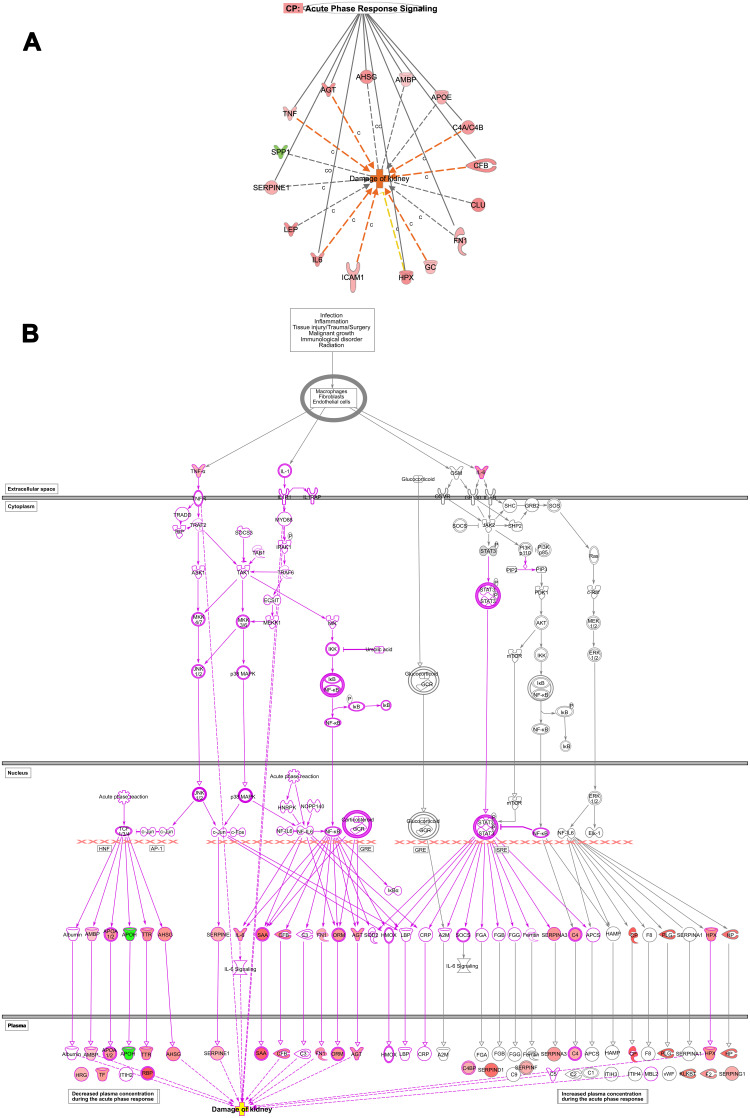
Renal diseases. (**A**) Networks display DEGs which is upregulated (red) and downregulated (green) genes involved in damage of kidney in T2DM based on the multiple corrections of the log P value. The deep orange arrow indicates the prediction of an increase of activation, yellow arrow indicates a decreased prediction of activation, and grey arrow could affect. IL6, ICAM1, GC, CFB, C4A/C4B, AGT, and TNF increase the prediction of kidney damage. (C), Causative and (CO), correlation relationships. (**B**) Network displays the role of acute inflammatory response pathway and cascade involving TNF and IL6 as an upstream regulator which affects the upstream transcription regulators; NFKB and NF-IL6 and kinases such as c-jun, and c-fos, which affects downstream targets related to damage of kidneys such as IL6, ICAM1, GC, CFB, C4A/C4B, AGT, and TNF which increase prediction of kidney damage.

We explore some specific disorders associated with organ injuries. Data obtained from the current analysis showed that 7 genes out of 16 which are CFB, AGT, IL-6, C4B, ICAM1, TNF, GC are increased, while HPX gene is decreasing, which have measurements consistent with predictive increases in damage of kidney with Z-score of 2.20, and a P value of 8.19^E-15^ (Figure 4A and B).

### Regulation of the Dysregulated Gene Expressions of the Data Set and Their Impact on Biological Functions and Diseases

#### Upstream Regulator Analysis of the Transcription Regulators and Kinases Factors

**Figure 5:**
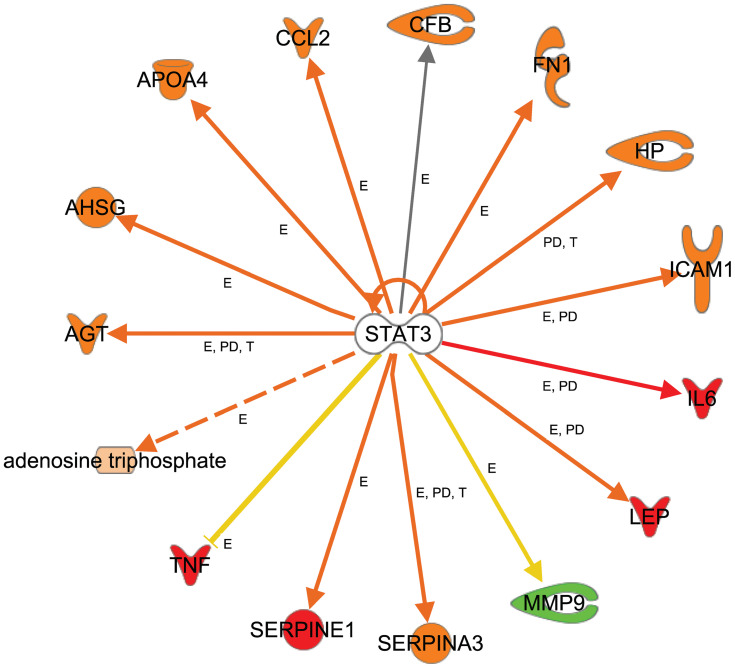
Upstream regulators-TF. Networks display target genes that are upregulated (red) and downregulated (green) genes in response to the top activated transcription factors as an upstream regulator in the data set of T2DM. Signal transducer and activator of transcription 3 (STAT3), relations in arrows are expression (E), protein–DNA interaction (PD), phosphorylation (P), and transcription (T). Arrows: brown, increase expression; golden arrow and dotted arrow, decreased; and grey, not predicted effect. Expression of HP, CCL2, LEP, SERPINA3, AGT, AHSG, ATP, APO4, SERPINE1, and ICAM1 is activated. The expression of MMP is inhibited. Others are affected but not predicted.

Further, we used the IPA upstream regulator analysis that would explain the changes in gene expression as downstream targets. The aim is to understand the underline regulation of the expression changes seen in the dataset of T2DM. Transcription factors are proteins that control the rate of RNA transcription to regulate the gene expression (up and down) based on the cell state and organ activity to help in cell homeostasis. The five most significant upstream transcript regulators based on *Z* score in the ranking were, Signal transducer and activator of transcription 3 (STAT3) as shown in Figure 5, Signal transducer and activator of transcription 1 (STAT1), Hypoxia-inducible factor 1-Alpha (HIF1A), followed by CCAAT/enhancer-binding protein alpha (CEBPA), followed by CCAAT/enhancer-binding protein beta (CEBPB) is presented in Table 8, and displayed in Supplementary figures (S3).

**Table 8:** Top Five Upstream Regulator Transcription Factors Affecting the Expression of the Genes of the Data Set of the Present Study with Their p-values, Z-Score, and Target Molecule (Based on Core Analysis in Ingenuity Pathway Analysis [IPA])

Name	Predicted Activation State	Activation z-Score	p-value of Overlap	Target Molecules	# of molecules Activated of the Data Set
STAT3	Activated	2.569	1.28^E−08^	AGT,AHSG,APOA4, ATP,CCL2, CFB,FN1, HP,ICAM1, IL6	10(15)
STAT1	Activated	2.521	1.28^E-11^	AGT,APOC2, APOE,C1R,C1S,C4A/C4B,CCL2, CFB,ICAM1, IL6	9 (15)
HIF1A	Activated	2.771	1.54^E−07^	AGT,APOE,ATP,FN1, HP,IL6, KRT14,LEP,MMP9,SERPINE1	9 (12)
CEBPA	Activated	2.046	3.12^E-10^	AGT,APOA4,APOB,APOC3,F9,HP,HPR,ICAM1,IL6,KRT14	9 (15)
CEBPB	Activated	2.288	3.85^E-16^	AGT,APOB,APOC3,CCL2,CP,FBLN1,HP,HPX,ICAM1,IGKC	11(20)

**Note:** Data represent active downstream target genes out of all downstream genes in parenthesis.

Further, we observed the top 5 activated kinases, which are ATM, JAK2, JAK1, MTOR, and AKT1, as upstream regulators that affect the expression of some genes of the data set as shown in Table 9, and supplementary Figure S4.

**Table 9:** Top Five Upstream Kinase Factors Affecting the Expression of the Genes of the Data Set of the Present Study with their p-values, Z-Score, Target Molecule and Numbers of Mechanistic Works. (Based on Core Analysis in Ingenuity Pathway Analysis [IPA])

Upstream Regulator	Predicted Activation State	Activation z-Score	p-value of Overlap	Target Molecules in Dataset	Mechanistic Network
ATM	Activated	2.607	2.16^E−08^	CLU, L6, LEP, NAD+, NADP, SERPINE1 (7)	40 (21)
JAK2	Activated	2.211	3.64^E−04^	CCL2, ICAM1, IL6, TF, TNF (5)	33 (19)
JAK1	Activated	2.189	8.77^E−06^	CCL2, ICAM1, LRG1, TF, TNF (5)	49 (24)
MTOR	Activated	2.187	2.86^E−03^	CCL2, FN1, IL6, LEP, MMP9, TNF (6)	54 (22)
AKT1	Activated	2.070	1.39^E-11^	APOC3,ATP,C1QC,CCL2,CLU,FN1,IL6,LEP,MMP9,PLG(14)	56 (23)

**Note:** Data in  brackets represent numbers of  direct mechanistic network affecting gene expression  out of all networks (direct and indirect).

**Table 10:** Top Regulator Proteins That Affect the Expression of Several Proteins of the Data Set of the Present Study with Their p-values, Z-Score, and Target Molecule (Based on Core Analysis in Ingenuity Pathway Analysis [IPA])

Top Regulator	Activation z-Score	p-value of Overlap	Target Molecules in Dataset
INS	0.967	2.15^E−09^	AGT,APOA1,ATP,GCG,GHRL,ICAM1,IGKC,IL6,LEP,TNF
LEP	2.185	8.71^E-14^	APOA1,APOA2,APOA4,APOH,CCL2,GHRL,HPX,ICAM1,IL6,INS,ORM1,phosphate,RETN, SERPINE1,SPP1,TN
IL6	3.175	1.81^E-20^	AGT,APOA1,APOB,APOE,ATP,CCL2,CLU,CP,FN1,GCG,HP,HPX,ICAM1,KRT14,LEP,LRG1, MMP9,ORM1,PLG,PON1,PPBP,SAA4,SERPINA3,SERPINE1,SPP1,TF,TTR

#### Top Regulator Effects

**Figure 6:**
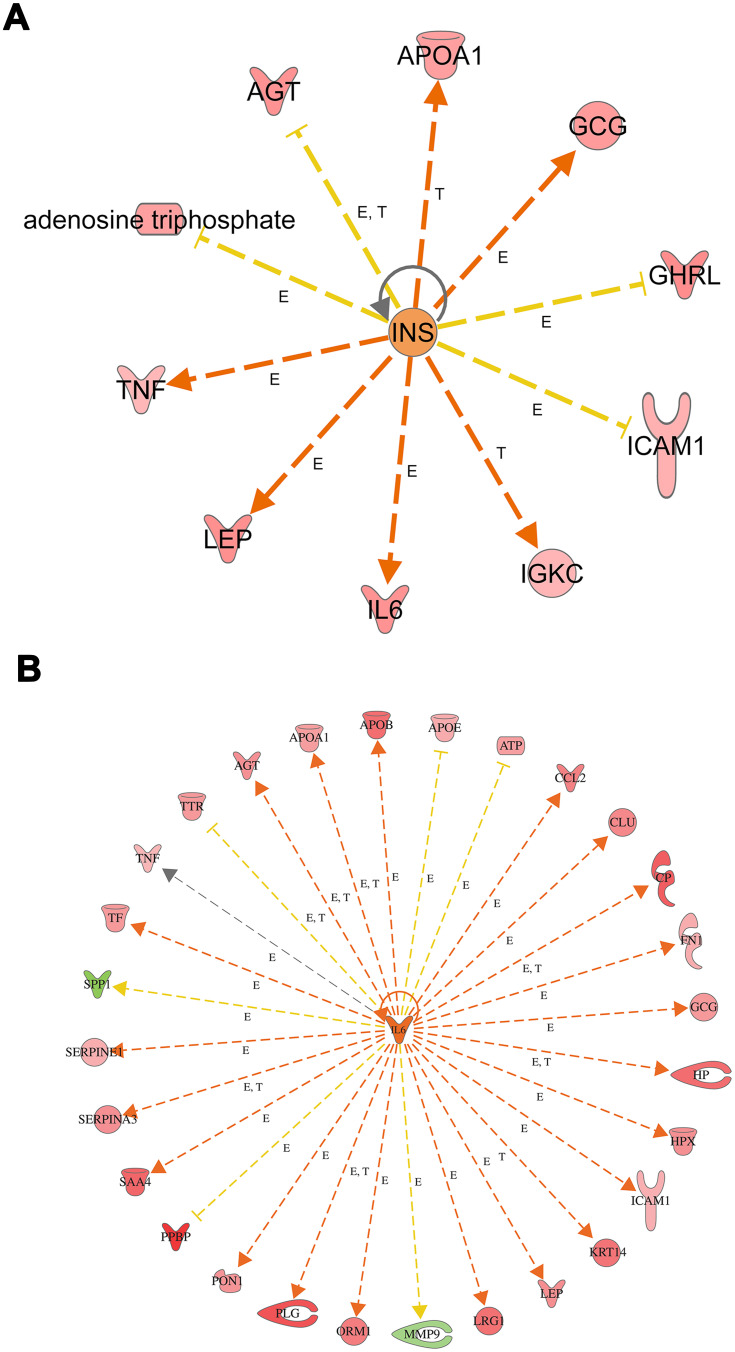
Top regulator effects (**A**) Regulator effects of the top downregulated proteins (INS) on target proteins of the dataset. Target genes that are upregulated (red) and downregulated (green) genes.  INS activates the expression of LEP, IL6, GCG, APOA1, IGKC, and TNF. INS inhibits expression of GHRL, AGT, ATP, and ICAM1. Brown, increased and golden arrow, decreased. Relations in arrows are expression (E), phosphorylation (P), and transcription (T). (**B**) Regulator effects of the top upregulated protein (IL6) on target proteins of the dataset. Target genes that are upregulated (red) and downregulated (green) genes. brown arrow: increase expression; golden arrow: decreased; and grey: not predicted effect. See Supplementary tables. Relations in arrows are expression (E), phosphorylation (P), and transcription (T).

Moreover, we look for the master regulator of proteins of the dataset, which regulate other proteins using IPA stream analysis. Also, this analysis identifies potential mechanisms linked with phenotype changes such as disease or functional disorders and explain the biological role of the upstream regulator, via its regulation on a gene or sets of genes. Table 10 illustrates the top three regulator proteins which regulate other proteins of the dataset. Among the top downregulated proteins of the dataset are, INS targets 10 molecules (Figure 6A), and SPP1 targets 9 of DEG (Figure S5a), while among the upregulated proteins are TNF targets 32, IL6 targets 29 (Figure 6B), LEP targets 19, AGT targets 14, and APOE targets 13 molecules of the data set (Table S11), and (Figure S5b)

#### Mechanistic Networks of Top Regulators

**Figure 7:**
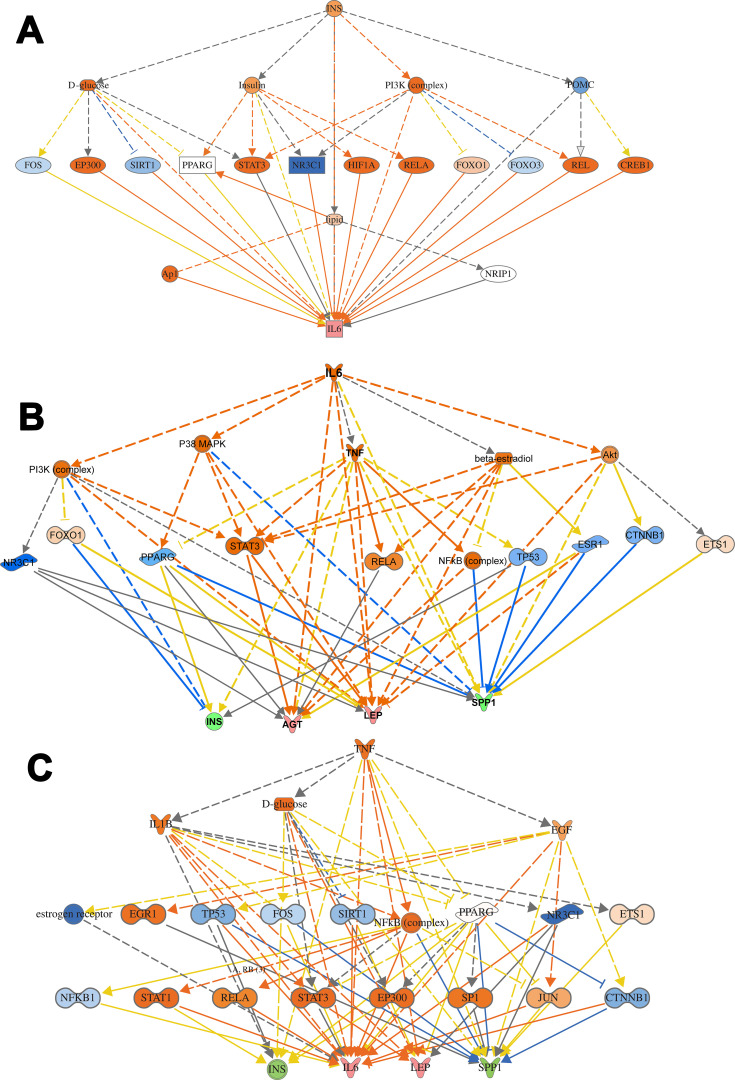
Mechanistic networks-protein interaction. (**A**) Networks display interactions of INS with some of the main target proteins of interest, such as IL6. The pointed arrowheads represent activating relationships, and blunt arrowheads represent inhibitory relationships. Trigger gene: INS; Upstream regulators: TFs; Target genes: IL6. (**B**) Network display interactions of IL6 with some of the main target proteins of interest such as INS, AGT, LEP, SPP1. The pointed arrowheads represent activating relationships, and blunt arrowheads represent inhibitory relationships. (**C**) Display interactions of TNF with some of the main target proteins of interest such as INS, IL6, LEP, and SPP1. Trigger gene: TNF; Upstream regulators: kinases and ligand nuclear receptors; Target genes: INS,IL6, LEP, and SPP1.

Further, in order to understand the mechanistic pathway by which a single protein affects a downstream target protein of the data set, we did the mechanistic analysis. For example, insulin is the primary hormone underlying the pathogenesis of T2DM and its comorbidities, which targets TNF, AGT, APOA1, GCG, GHRL, ICAM1, IGKC, IL6, and LEP. It was interesting to investigate the interaction of INS with some of the upregulated target proteins of interest such as TNF, IL6, and LEP, which regulate other proteins to understand the mechanistic of their interactions and crosstalk among a dataset of T2DM. INS upregulates LEP expression directly or indirectly through intermediates such as D-glucose, POMC, PI3 complex, which in turn affects transcription factors such as FOXO1, STAT3, SIRT1, EP 300, and via effects on ligand-dependent nuclear receptor such as PPARG, and NR3C1 (Figure S6). INS upregulates TNF gene expression directly or indirectly through intermediates such as D-glucose, SIRT1, EP 300, FOS, POMC, PI3 complex which in turn affects the transcription factors such as FOXO1, FOXO3, REL, STAT3, and via effects on ligand-dependent nuclear receptor such as PPARG, and NR3C1 and via the effect on lipid metabolism which acts directly or via c-JUN (Ap1) kinase (Figure S6). In similar basis, INS upregulates IL6 expression directly or indirectly (Figure 7A) through intermediates such as D-glucose, SIRT1, EP 300, FOS, POMC PI3 complex which in turn affects transcription factors such as FOXO1, FOXO3, REL, STAT3, and via effects on ligand-dependent nuclear receptor such as PPARG, and NR3C1 and via the effect on lipid which acts directly or via c-JUN (Ap1) kinase. The mechanical network analysis demonstrated two upregulated protein of the dataset., LEP targets 16 genes directly, and 28 genes indirectly of the data set via 21 possible mechanistic regulations (Figure S6), and IL6 targets 29 genes directly and 34 genes (Figure 7B) indirectly of the data set via 23 mechanistic regulators, TNF targets 32 genes directly, and 27 genes (Figure 7C) indirectly of the data set via 21 mechanistic regulators and one of the downregulated protein is INS which targets 10 genes directly and 34 genes indirectly via 20 mechanisms of mechanistic regulations.

**Figure 8:**
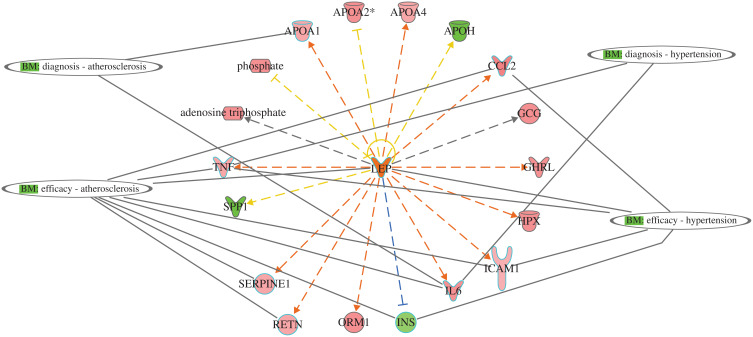
Network display utility of DRGs as predictive biomarkers for two comorbidities associated with T2DM: atherosclerosis and hypertension. APOA1 and IL6 as diagnostic for atherosclerosis, and CCL2, TNF, LEP, ICAM1, IL6, INS, SERPINE1, and RETN as efficacy biomarker for treatment. For hypertension, TNF and IL6 for diagnosis and CCL2, LEP, TNF, ICAM1, and IL6 as efficacy biomarkers for treatment.

The protein-protein interaction involves many hubs such as kinase, ligand-dependent nuclear receptor, transcription factor regulators, endogenous chemical, enzymes, and growth factors. As displayed in Figure (7B and C) for IL6 and TNF as the primary triggers of other proteins of the data set of T2DM. Such mechanistic interaction of proteins could explain the actions of such DRGs in diabetes and how it can modify insulin action on metabolic, cellular, and biological process/and disorders. (see supplement Figures S7a-d) and its uses as biomarkers in atherosclerosis and hypertension (Figure 8)

### Network Analysis

We investigated the interactions among the DRGs, pathways, regulators, and other molecules in type 2 diabetics and identified 12 eligible networks associated with various biologic processes, functions, and diseases-as displayed in a table (Table S12).

**Figure 9:**
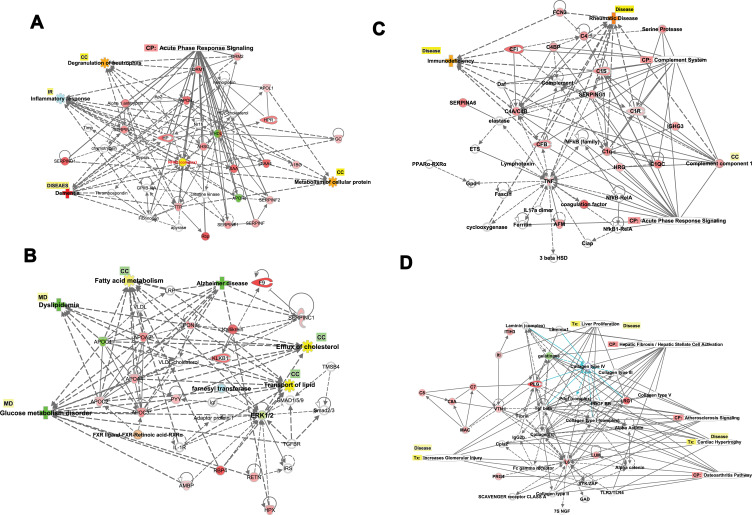
(**A**–**D**) Biological networks. The top four networks are showing interactions between dysregulated genes, functions, diseases, and upstream regulators in T2DM. Details are displayed in each figure. (**A**) NW1: The network displays cellular components, inflammatory responses, and neurological diseases. The network displays the canonical pathway and cellular components regulated by the DRGs. The upregulated (red) and downregulated (green) associated with dementia as a neurological complication of T2DM. (**B**) NW2: The network displays lipid metabolism, molecular transport, and small molecular biochemistry. The network displays the cellular functions and process (fatty acid metabolism, efflux of cholesterol) and inflammatory responses (IR) regulated by the DRGs. The upregulated (red) and downregulated (green) associated with dyslipidemia and glucose metabolic disorders and Alzheimer’s as neurological complications of T2DM. (**C**) NW3: The network displays developmental disorders, humoral immune response, and inflammatory responses. The network displays the canonical pathway and cellular process regulated by the DRGs. The upregulated (red) and downregulated (green) associated with immunodeficiency and rheumatoid arthritis as connective tissue disorders as a complication of T2DM. (**D**) NW4: The network displays cell to cell signaling and interaction cellular movements and inflammatory responses. The network displays the canonical pathway and cellular process regulated by the DRGs. The upregulated (red) and downregulated (green) associated with severe renal, hepatic, and cardiac injuries as complication of T2DM.

Network 1 included the top functions and diseases, which are Cellular Compromise, Inflammatory Response, and Neurological Disease, which comprise 16 DRGs. At the same time, the transcription factor NFKB complex is a hub for acute phase response signaling, cell functions such as degranulation, inflammatory response, and dementia as an example of a neurological disorder, as shown in (Figure 9A). Network 2 displays the top functions and diseases which are Lipid Metabolism, Molecular Transport, Small Molecule Biochemistry which comprise 14 DRGs; Apolipoproteins (APOC1, APOC2, APOC3, APOA2, APOA4) which are connected to the hubs of nuclear complexes NCOR-LXR-Oxysterol-RXR-9 cis linked to retinoid signaling activation, and FXR-ligand FXR-Retinoic linked to FXR-RXR activation and bile acid metabolism. The networks display proteins involved in blood hemostasis and coagulation such as F9, KLKB1, SERPINC1, HPX1, which crosstalk with each other and connected to Apolipoproteins via PON1. ERK1/2, which are extracellular signal-regulated kinases, acts as a hub, which is regulated by RBR4, RETN, and HPX, as shown in (Figure 9B). Network 3 displays the top functions and diseases, which are Developmental Disorder, Humoral Immune Response, Inflammatory Response, which comprise 13 DRGs. The network display two canonical pathways; the acute phase response signaling, which includes AFM, TNF, CFB, CRB, SERPING1, and CIS, and hub of NFKB family. The other crucial canonical pathway (CP) is the complement pathway, which includes genes such as CF1, CRB, SERPING1, CIS1, CIR, CFB, and complete component1 as a hub. The network displays the extensive crosstalk of TNF, SERPING1, and complement in network 3. Moreover, the figure display molecules involved in two crucial disorders associated with this network in T2DM, which are immunodeficiency and rheumatoid diseases (RD). In RD, many molecules are involved, such as complete component1, FCN, TNF, CFB, C1Q, and C1Q, as shown in Figure 9C. Network 4 displays the associated top functions and diseases, which are Cell-To-Cell Signaling and Interaction, Cellular Movement, Inflammatory Response, which comprise 9 DRGs. The top 3 CP are Hepatic Fibrosis, Atherosclerosis signaling, and GP6 signaling pathway. The immune response of macrophage as cell-to-cell signaling disorders involves 3 DRGs, which are IL6, LUM, VTN, the cellular movement disorders include IL6, VTN, and connective tissue disorders include collagen type11, IL6, LUM, PLG, and PRG4. Moreover, severe injury such as cardiac hypertrophy is displayed, which indicates the involvement of several DRGs of IL6, LUM, LRG1, PLG, with other molecules such as Alpha actinin, PDGF, Tgf beta, and TLR2 and TLR4. These toxic injuries of liver, heart, and kidney are severe complications in long-term and uncontrolled T2DM, as shown in Figure 9D. Other networks are involved in different diseases and functions related to T2DM and its comorbidities such as Cancer, Cardiovascular Disease, Organismal Injury, and Abnormalities (network 5).

## Discussion

The pathogenesis of T2DM and its associated comorbidities is presently challenging to identify specific biomarkers and pathways involved in its complications. Diabetes is a chronic polygenic disorder resulted from several biological processes that interact in a dense network, rather than from an abnormality of a single effector gene product. Since the biological functions are the results of molecular interactions, the functional annotations of differentially expressed genes should include the effect of many genes on different pathways and their interactions on the different biologic processes and networks that have a potential impact on T2DM. Understanding the biological pathways and their network information is useful in predicting the risk and understanding the progress of the disease using the integration of proteomics and metabolic dataset.

In the current study, IPA software was used to integrate the fold expression of DRGs for the development of molecular pathways and networks in T2DM. As illustrated in this study, the data explore significant pathways in T2DM subjects compared to control subjects to gain more understandings of the pathogenesis towards diabetic complications. This study identified differentially dysregulated genes as potential prognostic biomarkers involved in critical biological processes and pathways of proteins that are allied with T2DM comorbidities. The most important findings of this study are the identification of the upstream regulators which affect the gene expression such as transcription factors STAT3, STAT1, and HIF1A, cytoplasmic kinases such as JAK kinases and highlight their mechanistic actions that affect the expression. Furthermore, the findings of the present study identified the most commonly expressed genes: Tumor Necrosis Factor, TNF, Interleukin 6; IL6, Leptin; LEP, Angiotensinogen; AGT, Apolipoprotein E; APOE,Coagulation Factor II, Thrombin; F2, Secreted Phosphoprotein 1; SPP1,Resistin; RETN, and Insulin; INS that could be used as potential prognostic biomarkers. The data recognized that IL6 is the top regulator of the DRGs, followed by LEP. The study identified several networks which explore the dysregulation of several functions, including cell components, and molecular transport process associated with inflammatory responses that modify the insulin pathway. Top comorbidities and complications associated with DRGs are neurological and psychological disorders, organ injuries related disorders such as renal damage, and connective tissue disorders, and acute inflammatory disorders.

In this study, we identified 94 genes that were differentially dysregulated in Arab subjects with T2DM compared to healthy, non-diabetic controls. In order to explore the mechanisms causing changes in gene expression, we identified upstream regulators in order to provide biological insight into the observed expression changes. The essential top upstream regulator identified was IL6 of the data set, followed by transcription factors and kinases. IL6 is the master of all regulators, which controls 63 genes of the dataset, and it exerts its effects on the observed gene expression via 23 regulatory mechanisms with activation of Z-score (3.175). IL6 interacting directly on PI3 (Peptidase Inhibitor 3) complex, P38MAPK (stress signaler p38 mitogen-activated protein kinase), AKT (Serine/Threonine Kinase), and affects TNF gene expression and also through other regulatory molecules such as STAT3 (Signal Transducer And Activator Of Transcription 3), NFKBIB (NF-kappa-B inhibitor beta), and FOXO1 (forkhead box O1), (Figures 6 and 7B). The set of 23 regulators in total connects to the 63-dataset gene. IL6 is a growth factor with cytokine activity and protein binding. Interleukin 6 (IL-6), a multifunctional cytokine and has been linked to the pathogenesis of T2DM.^17^ Increasing the level of circulating IL-6 is a predictor biomarker of T2DM, especially in obese subjects and could be involved in the development of inflammation and insulin resistance.^18^

Furthermore, IL6 downregulates the expression of the insulin gene, which contributes to the pathogenesis of T2DM and its related comorbidities. IL6 is participating in numerous biological processes such as acute inflammatory response as one of the top canonical pathways associated with inflammatory response disease such as activation of leukocytes and degranulation of the cells, injury of different organs such as renal damage, connective tissue disorders such as rheumatoid arthritis. It also affects many molecular functions, such as fat and protein metabolism. The complex signal transduction mechanism of IL-6/STAT3 may explicate the widespread effects of the IL6 as a cytokine.^19^ Monitoring of IL6 in T2DM is of clinical significance as it is involved in many related complications such as atherosclerosis (Figure 8), hypertension (Figure 8), renal damage (Figure 4), metabolic, inflammatory disorders, and organs and tissues damage (Table 5).

Moreover, we detected several transcription factors (TF) as upstream regulators including Signal transducer and activator of transcription 3 (STAT3), Signal transducer and activator of transcription 1 (STAT1), Hypoxia-inducible factor 1-Alpha (HIF1A), CCAAT/enhancer-binding protein alpha (CEBPA), and CCAAT/enhancer-binding protein beta (CEBPB). Signal transducer and activator of transcription 3 is one of STAT family, known as acute-phase response factor, which regulates downstream target molecule such as; IL6, LEP, JAK2 (Janus Kinase 2), LIF (LIF Interleukin 6 Family Cytokine), IL10 (Interleukin 10), EGF (Epidermal Growth Factor), SRC (Proto-Oncogene, Non-Receptor Tyrosine Kinase), IL21 (Interleukin 21), SOCS3 (Suppressor Of Cytokine Signaling 3), JAK (Janus Kinase), IL6ST (Interleukin 6 Signal Transducer), and binds with other TFs such as EGFR (Epidermal Growth Factor Receptor), STAT1, JAK2 (Janus Kinase2), FOS (Fos Proto-Oncogene, AP-1 Transcription Factor Subunit), PIAS3 (Protein Inhibitor Of Activated STAT 3), IL6ST, SRC, EP300 (E1A Binding Protein P300), JUN (Jun Proto-Oncogene, AP-1 Transcription Factor Subunit), RELA (RELA Proto-Oncogene, NF-KB Subunit), JAK1, CDKN1A (Cyclin-Dependent Kinase Inhibitor 1A), HIF1A, STAT2. STAT3 is regulated by BCL2L1 (protein phosphatase 1, regulatory subunit 52), MYC (MYC proto-oncogene,), SOCS3, VEGFA (Vascular Endothelial Growth Factor A), IL6, CCND1 (Cyclin D1), STAT3, VEGF (Vascular Endothelial Growth Factor F), IL21, BIRC5 (Baculoviral IAP Repeat Containing 5), and HIF1A, which indicates its pleiotropic cellular effects such as expression, proliferation, apoptosis, growth, differentiation, and migration. In the current study, STAT3 regulates the expression of AGT, AHSG (Alpha 2-HS Glycoprotein), APOA4 (Apolipoprotein A4), ATP, CCL2 (C-C Motif Chemokine Ligand 2), CFB (Complement Factor B), FN1 (Fibronectin 1), HP (Haptoglobin), ICAM1 (Intercellular Adhesion Molecule 1), and IL6 (Table 8, Figure 5). The current findings of the role of STA3, and STAT1 as TFs, indicates its significant role in acute-phase response and inflammation, which is a hallmark of the biological process and related disorders associated with comorbidities of T2DM such as the damage of kidney which is enriched in the dataset. Previous studies indicated the role of STAT 3 protein in insulin resistance and diabetes and related disorders such as damage of kidney, degranulation of cells, and apoptosis of endothelial cells in microangiopathy.^20^ Such exploration of the role of the transcription factors and its downstream target genes could explain the numerous complications associated with T2DM.

Further, as upstream regulators that affect the gene expression of the dataset, we identified several kinases (Table 9, Figure S4), which can target several genes of the data set. AKT (Protein kinase B, PKB) is one of 3 closely related serine/threonine-protein kinases (AKT1, Akt2, and AKT3), which regulates metabolism, proliferation, cell growth, and angiogenesis.^21^ The dataset of the present study showed that AKT is regulated by insulin, EGF (Epidermal Growth Factor), PDGF (Platelet-Derived Growth Factor complex), TNF, and IGFI (Insulin-Like Growth Factor 1), and it regulates downstream several TFs such as GS3 B (Glutamine synthetase root isozyme B), NOS3 (Nitric Oxide Synthase 3), FOXO1 (Forkhead Box O1), mTOR (Mechanistic Target Of Rapamycin Kinase), and NFKB (Nuclear Factor Kappa B). It affects glucose metabolism function through translocation of the SLC2A4/GLUT4 (Solute Carrier Family 2 Member 4) glucose transporter to the cell surface post-insulin signaling effect.^21^,^22^ The analysis of the present findings of the current study demonstrated that the expression of the dysregulated gene is controlled by many factors such as transcription factors, cellular kinases, growth factors and cytokines such as IL6, TNF, and LEP, and all of them are involved in consequence of the biological process, pathways and diseases associated with T2DM, as we discussed further in next paragraphs.

The canonical pathway analysis by the core analysis of IPA (Figure 1) indicated several critical signaling pathways involved in the pathogenesis of T2DM and its comorbidities, such as Retinoid X Receptors (RXRs), and Acute Phase Response Signaling in the dataset of T2 DM (Table 3, and supplementary Tables S2–S7). RXRs are nuclear receptors that affect biologic functions such as lipid metabolism, molecular transport, and small molecule biochemistry. Activation of RXR is involved in cholesterol efflux in macrophages through an effect by the following DRGs of apolipoproteins family; APOE, APOC1, APOC2, APOC4, while inhibiting APOA5. In hepatocytes, activation of APOA4 enhances cholesterol efflux. The apolipoprotein family is transporters that are involved in lipid metabolism, which altered in diabetes and predisposed to several disorders such as atherosclerosis and cardiovascular diseases.^23^ Furthermore, TNF alpha is one of the DRGs which is involved in the transport of lipids, steroids, and efflux of cholesterol. TNF uses different signaling pathways such as NFKBIA, and P38MAPK to exert its effect on lipid transport.^24^ Such data are supported with the related functions observed in the present study such as fat acid metabolism, and lipolysis (Figure S2), and are interconnected to hypertriglyceridemia as a top disease related to metabolic disorder and considered as the leading platform and risk factor for multiple disorders such as cardiovascular disorders, and progressive neurological disorders (Table 5). The data of the present study revealed another nuclear receptor that is the Farnesoid X Receptor (FXR). The FXR is a member of the nuclear family of receptors and has emerged as a critical player in the control of numerous metabolic pathways such as a sensor of bile acid and its regulation (Figure 2). Bile acid receptor plays a vital role in fat and glucose metabolism.^25^ The activation of the membrane G-protein receptor 5 (TGR5) by bile acids is associated with metabolic actions such as ameliorating insulin resistance via GLP-1 secretion.^26^

Furthermore, the acute phase response pathway is one of the significant pathways observed in the current dataset. The pathway and its molecules are involved in inflammation included upregulated proteins via NFKB (Nuclear Factor Kappa B) as an upstream regulator, which is connected with 2761 nodes and could elucidate the plethoric effects of the acute phase response signaling pathway. One of these disorders associated with acute inflammatory response is the damage of the kidney. (Figure 4A and B) displays the pathway which identifies that TNF and IL6 are upstream regulators mainly via binding of TNF to TNF receptors at the plasma membrane where it activates a cascade of nuclear factors such as jun (Jun Proto-Oncogene), Ep300 (E1A Binding Protein P300), NF-kB (Nuclear Factor Kappa B), and NF-IL6 which in turn activates downstream targets of several proteins. The following up-regulated proteins as downstream targets are; Complement factor B; CFB [a marker of acute injury in sepsis],^27^ Angiotensinogen; AGT [a biomarker of diabetic nephropathy],^28^ Interleukin 6; IL6 [a biomarker of ischemic acute kidney injury, diabetic nephropathy, IgA nephropathy, and lupus nephritis],^29^ and Complement component 4, binding protein, alpha and beta (CA4/C4B) [IgA nephropathy], Intercellular adhesion molecule 1 (ICAM1) [a biomarker of glomerulonephritis],^30^ Tumor necrosis factor (TNF) [a biomarker of nephropathies, including immune complex-mediated glomerulonephritis],^31^ and Vitamin D Binding Protein (GC) [a biomarker of diabetic nephropathy],^28^ all are involved in the damage of the kidney.

Numerous essential metals are essential for the biological functions of several enzymes, proteins, and transcriptional regulators. It is also vital in many biochemical reactions for cell functions in different tissues. For instance, Zn, Mg, and Mn are cofactors of several enzymes that participated in various biological pathways. Zn is implicated in the biosynthesis and secretion of insulin hormone from the beta-cells of the pancreas. Likewise, Cr augments the activity of insulin receptors on muscle cells, which increases the insulin-stimulated glucose uptake.^32^ Several studies have described the pathogenic role of some essential metals might harmfully disturb pancreatic functions that lead to the development of diabetes.^32^ The data set of the current study showed that many DRGs is involved in increased prediction to amount of metal ions in T2DM such as CCL2 (C-C Motif Chemokine Ligand 2), F2 (Thrombin), FN1 (Fibronectin 1), GCG (Glucagon), GHRL (Ghrelin And Obestatin Prepropeptide), APOE, APOC3, AGT, TTR (Transthyretin), TNF, PYY (Peptide YY), PTH (Parathyroid Hormone), PPBP (Pro-Platelet Basic Protein), PLG (Plasminogen), ORM1 (Orosomucoid 1), ICAM1 (Intercellular adhesion molecule 1) as shown in (Figure 3). For example, TNF regulates the transport of divalent metals such as Zinc in cells, via the control of ZIP-importers and metallothionein gene expression.^33^

Complications observed with T2DM include disorders affecting the heart, blood vessels, nerves, eyes, liver, and kidneys.^34^ Hyperglycemia and insulin resistance have been reported as crucial players in the development of microvascular and macrovascular complications, including atherosclerosis.^35^ Overproduction of ROS species of endothelial dysfunction and inflammation precipitates the development of diabetic vascular disease.^36^,^37^ Close analysis of the data set (Figure S7b), shows the metabolite KNG1 (Kininogen 1) having a direct positive relationship of regulation for ROS generation,^38^ while FN1 (Fibronectin 1) regulates endothelial cell proliferation,^39^ F2 (Coagulation factor II) regulates endothelial cell function such as apoptosis^40^ and PLG (Plasminogen) negatively regulates inflammation.^41^ In attempting to monitor the onset of diabetic vascular disease, over-expression of KNG1, FNI, F2, and PLG could serve as prognostic biomarkers to monitor early vascular complications. Moreover, therapeutic applications to counteract vascular compromise, resolving of oxidative stress in T2DM patients could be achieved by regulating the expression of APOA1 which inhibits oxidative stress^42^ and RETN (resistin) which regulates the inflammatory response.^43^

Amyloidosis is a group of diseases in which misfolded proteins that result in progressive organ damage are formed in different tissues (Table 4). The clinical presentation depends on its location, and the liver, kidney, and heart are commonly affected (Table S9), causing liver cirrhosis, nephrotic syndrome, and heart failure, respectively.^44^ Several disorders were documented in the present study that dysfunction of the endothelium is considered as a crucial component in the pathogenesis of vascular disease including apoptosis, and oxidative stress (Figure S7b), which eventually leads to the development of diabetic-related complications such as atherosclerosis, diabetic retinopathy, and nephropathy.^45^ The damage of the genitourinary system includes several disorders such as urinary tract infection, abscess, chronic kidney disease, nephrotic syndrome, and urinary bladder, and urethral disorders.^46^ Disorders related to degranulation of cells, such as the chemotactic activity of neutrophils with a reduction in the phagocytosis and bactericidal activity (Figure S1) from diabetic patients is impaired, which predispose to infections .^47^ Further, we demonstrated increased activation of leukocytes (Table 5) with increased release of inflammatory sets of proteins such as IL6, and TNF, which could play an important role in inflammatory disorders (Figure 4A and B) associated with diabetes such as diabetic nephropathy .^48^

The coagulation pathway (Figure S7a) plays a critical role in the pathogenesis of cardiovascular disease in patients with neuropathy.^49^ Many diabetes patients die of cardiovascular complications. The examination of Figures S7a showed many metabolites that affect the coagulation in some fashion. INS regulates vascularization; APOH, F2, and ICAM regulate platelet activation and adhesion; AGT regulates blood vessel contraction; TNF and CCL2 regulate blood chemokine in circulation; F9 regulates onset Hemophilia B, and PON1 inhibits onset cardiovascular disease. Directions of therapeutic goals should aim at monitoring and decreasing over-expression of the metabolites contributing to coagulation diseases and increasing the counterattack of PON1 (Paraoxonase 1) to prevent cardiovascular morbidity.^50^ Other cellular processes plagued by T2DM patients include compromised wound healing, which is regulated by TNF and LEP metabolites; regulating their over-expression could resolve wound healing complications.^51^

Insulin action pathway networks identified dysregulation of cellular processes such as glucose and lipid metabolism, which plays a vital role in the pathogenesis of T2DM (Figure S7 b-d). Monitoring the coupled expression of dysregulated proteins such as IL6, INS, LEP, IAPP (Islet Amyloid Polypeptide), AGT, TNF, CCL2, GHRL, KNG1 (Kininogen 1), GCG, RETN, and C3 (Complement C3) could be used as a predictive biomarker of T2DM comorbidities. For instance, AGT (angiotensinogen) plays an essential role in counteracting both the cardiovascular and non-cardiovascular actions of AngII.^52^ Recent data have revealed that chronic administration of Ang 11 improves the action of insulin in glucose and lipid metabolism in obese mice.^53^ Potential mechanisms of this beneficial effect included activation of insulin signaling, inhibition of the adverse actions of AngII, and augmented transport of insulin to the target tissues.^54^,^55^

We observed an intersection between canonical signaling in T2DM and several DRGs that are tangled in neurological diseases, metabolic disorders, connective tissue disorders, immunodeficiency, and renal injuries associated with cellular functions and canonical pathways (Figure 9a-d). These communications are mediated through several factors, such as NFκB, Akt, ERK (extracellular signal-regulated kinases), TNF, and IL6. These factors were identified for their contributions in various diseases. Such networks indicate the complexity of the disorders associated with T2DM in terms of the target genes and their proteins on the different biological, cellular, and molecular functions which interact to cause a particular disorder. Even the complexity is evident in the current study as we identified in some examples that the upstream regulators affect abundant molecules such as growth factors like leptin; cytokines like TNF, and IL6; transcription factors such as HIFA, STAT, CEBPA, NFκB; and several kinases such as ERK, mTOR, AKT, and others.

Gathering information’s of the DRGs of the dataset, and their role in the various biological process with associated comorbidities in T2DM could help in understanding the pathogenesis of various disorders, enhance the utility of these genes as biomarkers for prediction, prognosis, complication and response to treatment, and design of new drugs that can be used in management.

Using the analysis of current data integration, we were able to answer some questions of interest. For example, TNF as one of the upstream regulators that are involved in rheumatoid arthritis, and the clinical utility of Adalimumab as antibody and binder to TNF target, could be used for the treatment of active rheumatoid arthritis, which is predicted to inhibit TNF as a target.^56^ Another question, which disease states could develop based on the activation of the acute phase response signalling signaling pathway in T2DM, the answer, for example, could be involved in the damage of the kidney, immunologic, and neurological disorders (Figures 4, 9A and C). STAT3 as an upstream regulator regulates 15 downstream target genes of the dataset and STAT3 could be involved in disorders related to inflammatory disorders such as connective tissues,^57^ vascular disorders such as atherosclerosis, angiogenesis,^58^ neurological disorders such as Alzheimer’s disorders^59^ and others based on the downstream target of the DRGs (Table 5, Figure 5). We analyzed the pathways, functions, diseases, and regulators to understand which gene or genes of that dataset could be used to monitor the prognosis, diagnosis, and efficacy of a particular disorder such as hypertension (IL6, TNF) for diagnosis of hypertension, while CCL2, IL6, LEP, and HPX for the efficacy of hypertension management, and, atherosclerosis and hemophilia (Figure 8, and Figure S7a). For example, ANG could be used to monitor the efficacy of Aliskiren and Irbesartan in the treatment of T2DM associated with renal disorders.^60^,^61^

In summary, the incorporation of metabolomics and proteomics data through integrative pathway analysis using different tools would help in understanding the role of various biomarkers in the identification of the biological processes, pathways, and pathophysiology that are associated with the comorbidities of T2DM. We identified the most commonly expressed genes of the study are the following TNF, IL6, LEP, AGT, APOE, F2, SPP1, RETN, and INS that could be used as potential prognostic biomarkers. The data recognized that IL6 is the top regulator of the DRGs, followed by LEP. LXR/RXR and acute phase response signalling pathways are dominant pathways involved in renal damage, insulin resistance, dyslipidemia, and cardiovascular disorders. We identified the role of upstream regulators such as STAT3 as a transcription factor that is involved in connective tissue disorders and atherosclerosis as it targets many proteins involved in such disorders. ANG could be used as an efficacy biomarker of renal disorders. TNF is involved in the regulation of metals such as Zinc, which affects B-cell functions and insulin secretion. Therefore, such information could help to recognize those patients at higher risk for a specific complication and its response to a particular class of anti-diabetic drugs. Prospective studies should be performed to validate the results obtained from the current work for the utility of the biomarkers using clinical studies.

A limitation of this study was a difference in the mean age of the control and diabetic: the age of the patents is older than the control, which could affect results. Therefore, we did an AUC analysis of some target biomarkers, as shown in supplementary Table 1, and previously published.^5^,^6^ The data showed that these markers were differentially expressed in the diabetic cohort, regardless of the age of the individual.
